# Dual Mode *p*HRI-*tele*HRI Control System with a Hybrid Admittance-Force Controller for Ultrasound Imaging

**DOI:** 10.3390/s22114025

**Published:** 2022-05-26

**Authors:** Teng Li, Xiao Meng, Mahdi Tavakoli

**Affiliations:** 1 Department of Electrical and Computer Engineering, University of Alberta, Edmonton, AB T6G 1H9, Canada; teng4@ualberta.ca; 2 Department of Mechanical Engineering, University of Alberta, Edmonton, AB T6G 1H9, Canada; xmeng1@ualberta.ca

**Keywords:** dual-mode control system, hybrid admittance-force controller, tele-operation, physical human–robot interaction, ultrasound imaging

## Abstract

The COVID-19 pandemic has brought unprecedented extreme pressure on the medical system due to the physical distance policy, especially for procedures such as ultrasound (US) imaging, which are usually carried out in person. Tele-operation systems are a promising way to avoid physical human–robot interaction (*p*HRI). However, the system usually requires another robot on the remote doctor side to provide haptic feedback, which makes it expensive and complex. To reduce the cost and system complexity, in this paper, we present a low-cost, easy-to-use, dual-mode *p*HRI-teleHRI control system with a custom-designed hybrid admittance-force controller for US imaging. The proposed system requires only a tracking camera rather than a sophisticated robot on the remote side. An audio feedback is designed for replacing haptic feedback on the remote side, and its sufficiency is experimentally verified. The experimental results indicate that the designed hybrid controller can significantly improve the task performance in both modes. Furthermore, the proposed system enables the user to conduct US imaging while complying with the physical distance policy, and allows them to seamlessly switch modes from one to another in an online manner. The novel system can be easily adapted to other medical applications beyond the pandemic, such as tele-healthcare, palpation, and auscultation.

## 1. Introduction

The COVID-19 pandemic has posed a huge challenge to medical systems due to the physical distance policy [1], especially for medical procedures that usually require physical contact, such as injections, palpation, and ultrasound (US) imaging. This has led to a growing interest in robotics in the fields of medicine and healthcare, such as robot-assisted systems, automated control systems, and tele-operation systems, which are regarded as promising substitute methods when physical human–robot interaction (*p*HRI) is limited or not available [1,2,3,4,5].

US imaging is widely used in the medical field, even including dentistry, due to its characteristics of being non-invasive, inexpensive, and radiation-free [6,7]. Traditionally, US imaging relies on sonographers to physically move the US probe on the patient’s body. A robotized method is to attach the probe to a robot end-effector (EE) for assistance [8,9]. For example, Carriere et al. [8] designed an admittance-controlled robot-assisted system for US scanning. Their system enabled the robot to automatically control the probe orientation and the probe–tissue contact force while the user controlled the lateral position of the US probe. A three-dimensional (3D) scanner was required to reconstruct the 3D surface of the soft tissue in real time in their system. Fang et al. [9] developed a cooperatively controlled robotic system to enable adaptive contact force assistance by involving a dual force sensor setup. Their system was demonstrated to have the ability to reduce user effort and improve image stability. Akbari et al. [10] developed an image quality online assessment algorithm for US scanning systems with which the system can automatically adjust the contact force. Soleymani et al. [11] designed a 3D-printed US scanning mechanism that enabled the operator to perform the US imaging task two meters away from the patient. A survey on robotic US systems in medicine can be found in [12].

In order to respect physical distancing, i.e., to avoid physical interaction, tele-operation systems could be an alternative, promising solution for US imaging. There is a long history in the use of tele-operation systems for US imaging even before the pandemic [12,13,14,15,16,17,18]. Two decades ago, Mitsuishi et al. [16] developed a remote US diagnostic system. Their tele-operation system consists of a 6-degree-of-freedom (DOF) leader manipulator attached with a three-axis force sensor on the doctor side, and a 7-DOF follower manipulator attached with another three-axis force sensor on the patient side. To ensure safety, their US probe can be retracted at any time to avoid injuring the patient. Their leader and follower manipulator has a distance as far as approximately 700 km.

Conti et al. [19] presented a tele-operation robotic system to assist sonographers in conducting US imaging in aiming to reduce physical fatigue. Their system utilized a 7-DOF Kuka LWR robot as the follower on the patient side and a 6-DOF haptic device as the leader on the doctor side. The system allows users to remotely operate the follower with force feedback, while the contact force remains at a pre-defined level.

Most recently, Duan et al. [18] developed a tele-operated robotic system for remote US diagnosis. Their system has a set of sophisticated control consoles on the doctor side, which can be used to remotely control the robot on the patient side to conduct US imaging.

The traditional tele-operation system is able to provide relatively accurate position/force control and realistic haptic feedback [16,19]. However, as introduced above, it usually requires a second robot and/or a sophisticated control panel to be deployed on the remote side to establish a leader–follower system, which could make the system expensive and complicated to install and operate. This is also viewed as a major factor that hinders the popularity of tele-operation systems in the healthcare industry [19]. Moreover, many other challenges, such as control algorithm complexity and controller stability, must be dealt with during the development of such a leader–follower system.

**Figure 1:**
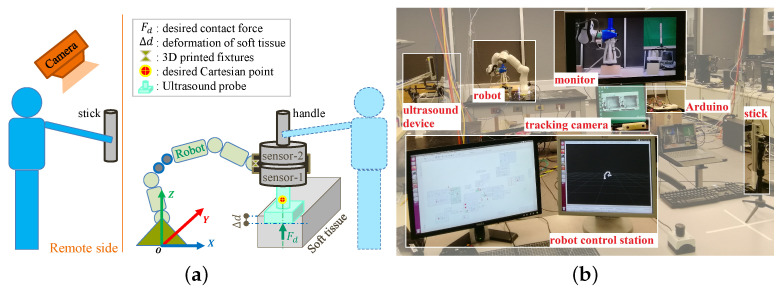
Schematic setup and real scene for the proposed dual-mode *p*HRI-teleHRI control system. Note that sensor-1 and sensor-2 are external force/torque sensors of the same type, and they are stacked and installed by using a specially designed fixture to ensure that the two sensors work independently without affecting each other. In *p*HRI mode, the user can directly apply force on the handle attached at the robot EE in order to move the robot EE. In teleHRI mode, the user will hold and move the stick in order to move the robot EE while the stick pose is tracked by a tracking camera in real time. (**a**) Schematic setup. (**b**) Real scene.

In aiming for a low-cost, easy-to-use system, in this paper, a novel dual-mode *p*HRI-teleHRI control system with a custom hybrid admittance-force controller is developed, as illustrated in Figure 1. The proposed system needs only a tracking camera on the remote doctor side, which can largely reduce the cost and system complexity. During the development of such a system, a major challenge is the feedback design, since haptic feedback is not available on the remote doctor side. To address this issue, a concise audio feedback is designed to indicate the real-time contact force status, and its sufficiency for replacing haptic feedback is experimentally verified. Then, the effectiveness and robustness of the proposed dual-mode system are experimentally evaluated.

The remaining parts of this paper are organized as follows: Section 2 describes the methods in detail, including the dual-mode control system design, controller design, camera–robot mapping algorithm, and audio feedback design. Section 3 presents experiments and corresponding results for developing and evaluating the proposed control system. Section 4 provides some discussions on the results. Section 5 presents the concluding remarks.

## 2. Methods

This paper describes an experimental study, where we first develop a dual-mode control system and then evaluate its effectiveness experimentally. In this section, we introduce the design methodology of the proposed dual-mode system in detail. First, the robot dynamics are described. Then, the admittance control used in *p*HRI mode is elaborated, followed by the mapping algorithm between the robot and the tracking camera, which will be implemented in teleHRI mode. The force controller is then introduced, which will be employed in both modes. Lastly, we introduce the apparatus and audio feedback design that will be used for subsequent physical experiments.

### 2.1. Robot Dynamics

The general dynamic model for an *n*-degree-of-freedom (DOF) rigid robot [20] can be given by

(1) $$M\left(q\right)\ddot{q}+S\left(q,\dot{q}\right)\dot{q}+g\left(q\right)=\tau +J^{T}F_{\mathrm{ext}}$$

where $M\in R^{n\times n}$ denotes the inertia matrix, $S\in R^{n\times n}$ denotes a matrix related to the Coriolis and centrifugal forces, $g\in R^{n}$ represents a gravity related vector, $\tau \in R^{n}$ is the commanded joint torque vector, $F_{\mathrm{ext}}\in R^{6}$ is the external force in Cartesian space (e.g., robot–human interaction force, robot–environment contact force), and $J\in R^{6\times n}$ is the Jacobian matrix.

As shown in (1), the robot can directly receive and execute torque command τ if a torque control interface is available. For some commercial robotic manipulators, however, interfaces for direct torque control may not be provided by the manufacturers due to safety issue considerations or other reasons. Instead, a velocity and/or position control interface is commonly provided. In this scenario, torque-related control methods such as impedance control [21] will not be usable. Alternatively, an admittance controller can be implemented on top of the velocity/position control interface in order to enable physical human–robot interaction.

### 2.2. Admittance Controller for pHRI Mode

The general mass–spring–damper model for admittance control in Cartesian space [21,22] can be expressed as

(2) $$F_{h}=M_{1}{\ddot{x}}_{\mathrm{ad}}+D_{1}{\dot{x}}_{\mathrm{ad}}+K_{1}x_{\mathrm{ad}}$$

where $F_{h}\in R^{6}$ is the external force applied on the robot EE (e.g., by the human user), and $M_{1}$, $D_{1}$, $K_{1}\in R^{6\times 6}$ are the coefficient matrices of mass, damper, and spring, respectively, and need to be designed. $x_{\mathrm{ad}},{\dot{x}}_{\mathrm{ad}},{\ddot{x}}_{\mathrm{ad}}$ are the relative position, velocity, and acceleration, respectively, in Cartesian space, which are caused by $F_{h}$.

For admittance control, the input signal in the mass–spring–damper model (2) is the external force $F_{h}$, while the output signal is the relative position displacement $x_{\mathrm{ad}}$ (or the relative velocity ${\dot{x}}_{\mathrm{ad}}$ when the spring term is removed). Taking the relative displacement $x_{\mathrm{ad}}$ as the output and rewriting (2), the transfer function from $F_{h}$ to $x_{\mathrm{ad}}$ for an admittance controller can be expressed in the time domain as

(3) $$x_{\mathrm{ad}}=K_{1}^{-1}\left[F_{h}-M_{1}{\ddot{x}}_{\mathrm{ad}}-D_{1}{\dot{x}}_{\mathrm{ad}}\right]$$

It can be simplified as (4) when the spring term $K_{1}x_{\mathrm{ad}}$ is disabled.

(4) $${\dot{x}}_{\mathrm{ad}}=D_{1}^{-1}\left[F_{h}-M_{1}{\ddot{x}}_{\mathrm{ad}}\right]$$

In this paper, Equation (4) will be used as the transfer function of the admittance controller in the *p*HRI mode. Without involving the spring term in (4), the robot will not recover the initial position after the external human force is removed.

**Figure 2:**
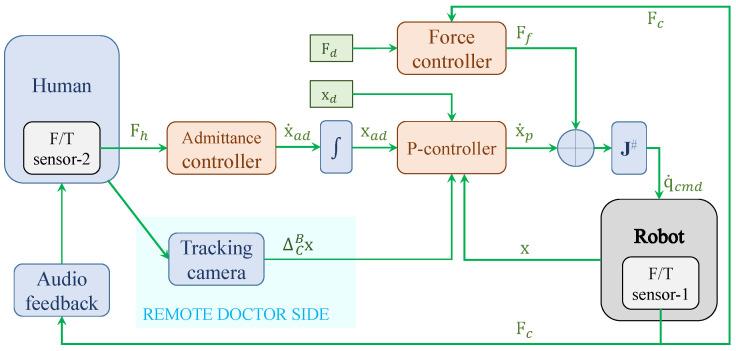
Block diagram of the proposed dual-mode *p*HRI-teleHRI control system with a custom hybrid admittance-force controller. $F_{c}$ represents the probe–tissue contact force measured by sensor-1, $F_{h}$ represents the external human force measured by sensor-2, $F_{f}$ represents the output of the force controller, ${\dot{x}}_{p}$ represents the generated Cartesian velocity from the P-controller, x represents the actual pose of the robot EE in Cartesian space, $J^{\#}$ represents the pseudoinverse of the Jacobian matrix, and ${\dot{q}}_{\mathrm{cmd}}$ represents the joint velocity command sent to the robot.

In summary, the procedures of admittance control used in this paper are as follows. As illustrated in Figure 2, first, an external human force $F_{h}$ is applied on the robot EE and measured by a force/torque (F/T) sensor. Then, $F_{h}$ is converted into a relative velocity ${\dot{x}}_{\mathrm{ad}}$ via the admittance controller (4). Then, the output velocity ${\dot{x}}_{\mathrm{ad}}$ from the admittance controller is converted into Cartesian displacement $x_{\mathrm{ad}}$ by an integrator. Finally, the displacement $x_{\mathrm{ad}}$ is added onto the initial desired robot EE pose $x_{d}$ in a P-controller. Therefore, the *p*HRI mode is established based on the admittance controller (4).

### 2.3. Mapping Algorithm for teleHRI Mode

Besides the *p*HRI mode from the admittance controller, another teleHRI mode is designed for remote operation. As mentioned earlier, only a tracking camera and a stick (with a marker attached) are needed on the remote doctor side.

Assume that the tracking camera frame on the remote doctor side is denoted as {C} and the robot base frame on the patient side is denoted as {B}. A direct frame mapping method between the two frames is established by

(5) $$\left\{\begin{array}{c} {}^{B}x={}_{C}^{B}T{}^{C}x\\ {}_{C}^{B}T=\left[\begin{array}{cccccc} 0 & 0 & -1 & 0 & 0 & 0\\ 1 & 0 & 0 & 0 & 0 & 0\\ 0 & -1 & 0 & 0 & 0 & 0\\ 0 & 0 & 0 & 0 & 0 & -1\\ 0 & 0 & 0 & 1 & 0 & 0\\ 0 & 0 & 0 & 0 & -1 & 0 \end{array}\right] \end{array}\right.$$

where ${}^{B}x$ and ${}^{C}x$ are the poses of the stick in the robot base frame {B} and in the camera frame {C}, respectively. ${}_{C}^{B}T$ is the direct transformation matrix from {C} to {B}. Please note that in (5), ${}_{C}^{B}T$ can be customized as necessary, and the main advantage of using the direct frame mapping method here is the robustness compared with using a 4-by-4 homogeneous transformation matrix, which requires accurate rotating angles and translations.

Based on the mapping method (5), a relative-displacement-based mapping algorithm between the pose of the stick (on the remote doctor side) and the pose of the robot EE (on the local patient side) is designed, and it can be expressed as

(6) $$\left\{\begin{array}{c} {}^{B}x_{d}={}^{B}x_{d}+\Delta {}_{C}^{B}x\\ \Delta {}_{C}^{B}x={}_{C}^{B}T\Delta {}^{C}x\\ \Delta {}^{C}x={}^{C}x-{}^{C}x_{0} \end{array}\right.$$

where ${}^{B}x_{d}\in R^{6}$ is the desired pose of the robot EE (attached with US probe) in the robot base frame {B}, $\Delta {}_{C}^{B}x\in R^{6}$ is the relative displacement of the stick in tracking the camera frame {C} as expressed in the robot base frame {B}, ${}^{C}x\in R^{6}$ and ${}^{C}x_{0}\in R^{6}$ are the real-time pose and the initial pose of the stick in the camera frame {C}, respectively. Note that the first equation in (6) describes the updating rule for the desired robot EE pose (${}^{B}x_{d}$) based on the real-time relative displacement of the stick ($\Delta {}_{C}^{B}x$).

### 2.4. Force Controller

The general form of the Cartesian space force tracking controller [23] can be given by

(7) $$F_{f}=K_{p}\left(F_{c}-F_{d}\right)+K_{i}\int_{0}^{t}\left(F_{c}-F_{d}\right)\mathrm{dt}+K_{d}\left({\dot{F}}_{c}-{\dot{F}}_{d}\right)$$

where $K_{p},K_{i},K_{d}\in R^{6\times 6}$ are the designed proportional, integral, and derivative coefficient matrices, respectively, in Cartesian space, and are typically diagonal. $F_{d},F_{c}\in R^{6}$ are the desired and actual contact force between the robot EE and environment, respectively. For simplicity, in this paper, a PI force controller is employed, as given by

(8) $$F_{f}=K_{p}\left(F_{c}-F_{d}\right)+K_{i}\int_{0}^{t}\left(F_{c}-F_{d}\right)\mathrm{dt}$$

where the actual contact force $F_{c}$ is measured by an external F/T sensor.

Combining the admittance controller (4) for *p*HRI mode, the mapping algorithm (6) for teleHRI mode, and the force controller (8) together, a hybrid admittance-force controller for a dual-mode *p*HRI-teleHRI control system is constructed. The block diagram for the proposed dual-mode control system is shown in Figure 2, while the corresponding setup is illustrated in Figure 1a.

### 2.5. Apparatus

A 7-DOF Franka Emika Panda robot (Franka Emika GmbH, Munich, Germany) is used for developing the proposed dual-mode *p*HRI-teleHRI control system, as shown in Figure 1. The proposed control system is implemented on the Panda robot via a joint velocity control interface and MATLAB/Simulink (version R2019a, MathWorks Inc., Natick, MA, USA) code. The Simulink runs on a workstation computer of Intel(R) Core(TM) i5-8400 CPU @ 2.80 GHz × 6 with the Ubuntu 16.04 LTS (Xenial Xerus) 64-bit operating system. The control rate of the Panda robot is 1000 Hz.

A commercial MicronTracker with interface library MTC 3.8 (ClaroNav Inc., Toronto, ON, Canada) is used as the tracking camera on the remote doctor side to track the pose of the stick in real time with a frequency of 20 Hz. Please note that the commercial MicronTracker can be replaced by a commonly used regular camera for a lower cost.

In this paper, the contact force between the US probe and tissue is measured by a 6-DOF F/T sensor (sensor-1 in Figure 1, Axia80-M20-ZC22, ATI Industrial Automation, Inc., Apex, NC, USA). In the meantime, a second F/T sensor (sensor-2 in Figure 1) of the same type is used to measure the external interaction force exerted on the robot EE handle by the human user, thus indicating user effort. The two sensors are stacked together as illustrated in Figure 1a, and an exclusively designed fixture for mounting the two sensors on the robot EE is used to ensure that the two sensors work independently.

### 2.6. Audio Feedback and Haptic Feedback

Audio, visual, and haptic feedback are the most commonly used feedback types in research. As a potential replacement for haptic feedback, audio feedback (AF) is selected as a low-cost and simple alternative. Audio feedback is designed to indicate the real-time contact force status between the US probe and the soft tissue.

The normal contact force between the US probe and the tissue during scanning is one of the most important indicators since a stably controlled contact force can guarantee the US image quality [8,19]. Different clinical examination types usually involve different desired ranges of contact force—for example, a general range of 5–20 N for cardiac, renal, and abdominal examinations [24,25], and 6.4 N for carotid examinations [26]. Empirically, in this paper, acceptable image quality can be obtained when the contact force is around 4.5 N. Therefore, an a priori decision is made to use a desired range of 4.5±1 N for the normal contact force under the assumption that it can ensure high-quality scanning images. Note that the force controller in the proposed system is capable of setting other constant or varying desired forces.

Based on the desired force range, audio feedback is designed to indicate which range the current normal contact force is located in, i.e., lower range [−inf,3.5] N, desired range [3.5,5.5] N, and upper range [5.5,+inf] N. Audio feedback is provided in all experiments via an Arduino board and a buzzer. A continuous beep sound is used to indicate the desired range, while a discontinuous fast beep is used to indicate the upper range. Otherwise, no audio feedback is provided. In detail, the audio feedback signals are generated by supplying 5V DC signals from a classical Arduino MEGA2560 (R3) board to a passive buzzer (OSOYOO TMB12A05). The designed audio signals are given by

(9) $$h\left(t\right)=\left\{\begin{array}{cc} 0\; & F_{z}\in \left(-inf,3.5\right)\\ h_{1}\; & F_{z}\in \left[3.5,5.5\right]\\ h_{1}\times a\times f\left(Freq,t\right)\; & F_{z}\in \left(5.5,+inf\right) \end{array}\right.$$

where h(t) is the generated time-related audio signal, $h_{1}=20$ is the selected factor for pulse width modulation (PWM), $F_{z}$ is the real-time normal contact force between the US probe and tissue, a×f(Freq,t) is a function of square wave form with respect to time *t* with setting Freq=8,a=3.

On the other hand, haptic feedback (HF) is presented in *p*HRI mode. Please note that haptic feedback in this work refers to the natural haptic feedback when physical human–robot interaction occurs, rather than specially designed feedback.

## 3. Experiments and Results

### 3.1. Procedures and Metrics

**Figure 3:**
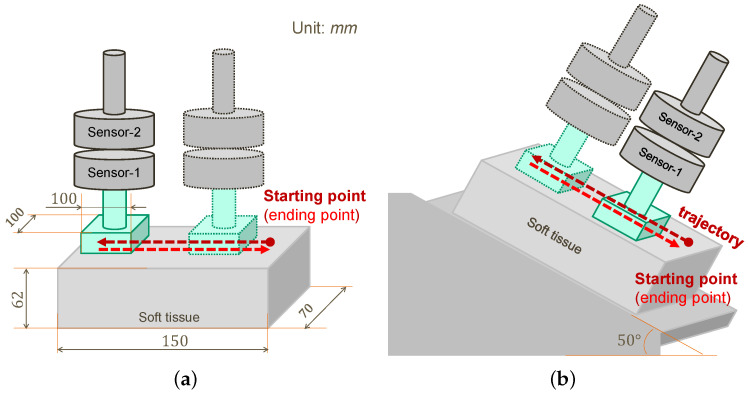
Two scenarios of soft tissue surface, i.e., horizontal and inclined slope. (**a**) Horizontal scenario. (**b**) Slope scenario.

There are three experiments designed in this section for developing and evaluating the proposed dual-mode *p*HRI-teleHRI control system. In particular, Experiment 2 involves two tissue surface scenarios of horizontal and slope, as shown in Figure 3. All experiments employ the same procedure for performing the ultrasound (US) imaging task, i.e., 6 sessions are required in each mode (*p*HRI or teleHRI) while each session includes 3 trials. One trial is defined as the US probe moving over the soft tissue surface, starting at one end, moving to the other end, and then returning to the starting point. The *p*HRI mode represents the physical human–robot interaction method established by an admittance controller, while the teleHRI mode represents the tele-operation method established by a tracking camera system.

The performance metrics involved in the experiments are listed as follows:

- Normal contact force, mean and variance (squared standard deviation) between the US probe and the soft tissue. The former indicates task performance accuracy while the latter indicates task performance stability.
- User effort, in units of Newton. It is indicated by the force exerted on the robot EE by the human user in *p*HRI mode, and also serves as input signals for the admittance controller. It is measured by sensor-2, as shown in Figure 1.
- Time percentage. A percentage for retaining the normal contact force within the desired range in one trial.

In the admittance controller (4), the coefficient matrices $M_{1}$ and $D_{1}$ are parameterized as $M_{11}=0.01I_{3\times 3}$ and $D_{11}=14I_{3\times 3}$ for the translational part and $M_{22}=0.0001I_{3\times 3}$ and $D_{22}=1.5I_{3\times 3}$ for the orientational part. For simplicity, the US probe is assumed to be exactly perpendicular to the tissue surface during the task (a more sophisticated 3D soft tissue reconstruction method may be required for cases beyond this assumption [8]); then, the normal contact force can be measured by the *z*-axis of the F/T sensor directly in the sensor frame. In the force controller (8), the desired force is set as $F_{d}=\left[0,0,4.5,0,0,0\right]$ in the F/T sensor frame and then transformed into the robot base frame. A *t*-test is employed for statistical analysis and a *p*-value of 0.05 is adopted as the significance level. Video S1 demonstration for the experiments is available online https://drive.google.com/file/d/1rwz_5fpUVSh2QEDMGiJansBVoiGXbDpO/view?usp=sharing accessed on 12 December 2021.

### 3.2. Experiment 1: AF vs. AF + HF

In Experiment 1, audio feedback and haptic feedback (AF + HF) are presented in *p*HRI mode while audio-only feedback (AF) is presented in teleHRI mode during the US imaging task. In this experiment, we investigate how different feedback affects task performance. No force controller is implemented in this experiment, which means that both the lateral movement of the US probe and the normal contact force are controlled by the user.

This experiment requires the user to perform the US imaging task on a horizontal tissue surface in *p*HRI mode and teleHRI mode, respectively, with different feedback. As described earlier, a total of six sessions are required in each mode, while each session includes 3 trials. During the task, the user needs to manually control the lateral movement of the US probe and also needs to maintain the normal contact force between the the probe and the tissue in the desired range. As mentioned earlier, the task performance accuracy is indicated by the mean normal contact force throughout this paper, while the task performance stability is indicated by the corresponding variance.

**Figure 4:**
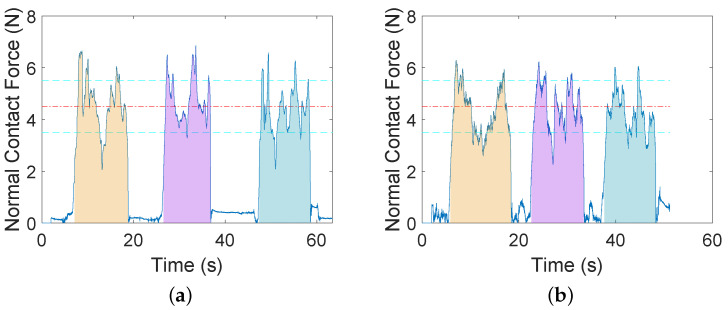
Sample data for US scanning on horizontal tissue surface with different feedback are provided in two modes in Experiment 1. AF means audio feedback; HF means haptic feedback. Two horizontal dashed cyan lines define the tolerance area for the desired force. (**a**) AF + HF (*p*HRI mode). (**b**) AF (teleHRI mode).

Statistical analysis on the results (see Table A1 for details) shows that there is no significant difference (p=0.5457) in the mean normal contact force between the two modes, but there is a significant difference (p=0.0420) between their variances, which means that the human user has significantly more stable task performance (i.e., smaller variance) with AF in teleHRI mode than with AF + HF in *p*HRI mode. A sample of data is presented in Figure 4. As can be seen in the figure, the normal contact force cannot stably remain in the desired range in either mode. This is also reflected by the time percentages for retaining the force in the desired range (see Table A2 for details), which are lower than 75% in both modes (58.75% in *p*HRI mode and 74.37% in teleHRI mode).

The results from Experiment 1 show that the task performance accuracy in teleHRI mode with AF is comparable to that in *p*HRI mode with AF + HF in terms of averaged normal contact force. The task performance stability in teleHRI mode with AF is significantly better than that in *p*HRI mode with AF + HF in terms of their variances. These results indicate that the audio-only feedback (AF) is as good as audio-haptic feedback (AF + HF); thus, the audio feedback is able to serve as a replacement for the haptic feedback in our case.

### 3.3. Experiment 2: AF + FC vs. AF + FC + HF

In Experiment 2, the hybrid admittance-force controller is implemented. More specifically, an additional force controller (FC) is implemented into the control system in both *p*HRI and teleHRI modes based on Experiment 1. This means that the normal contact force is regulated by the robot FC while the lateral movement is controlled by the human user in Experiment 2. As a further step based on Experiment 1, this experiment investigates how the different feedback will affect task performance when the proposed hybrid controller is implemented. The task procedures are the same as those described in Experiment 1. In particular, two tissue surface scenarios, namely the horizontal scenario and slope scenario (Figure 3), are considered in Experiment 2 in order to test the flexibility of the proposed system.

(1) Experiment 2a: Horizontal scenario

In Experiment 2a, the US imaging task is conducted on a horizontal soft tissue surface. Statistical analysis (see Table A3 for details) shows that the task performance accuracy in teleHRI mode with AF + FC is significantly better than in *p*HRI mode with AF + FC + HF ($p=2.6999\times 10^{-4}$) in terms of mean normal contact force. Despite this significance, it is worth noting that the max–min magnitude difference on the normal contact force across all sessions is only 0.17 N, which is close to the F/T sensor resolution 0.1 N. There is no significant difference between their variances in the two modes (p=0.1755), which indicates that the task performance stability in the two modes is comparable.

**Figure 5:**
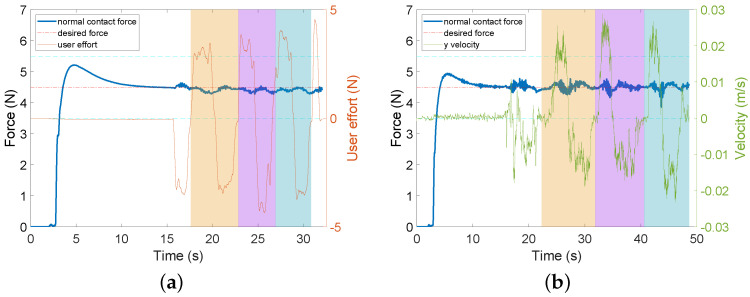
Sample data for US scanning on horizontal tissue surface with different feedback are provided for two modes in Experiment 2a. AF means audio feedback; HF means haptic feedback; FC means force controller. Two horizontal dashed cyan lines define the tolerance area for the desired force. (**a**) AF + FC + HF (*p*HRI mode). (**b**) AF + FC (teleHRI mode).

A sample of data for Experiment 2a is shown in Figure 5. As can be seen from the figure, user effort in *p*HRI mode is in the range of [−5,5] N, which indicates that the user can easily control the lateral movements of the US probe when an additional force controller is implemented.

(2) Experiment 2b: Slope scenario

In Experiment 2b, the US imaging task is conducted on an inclined slope soft tissue surface in *p*HRI mode and teleHRI mode separately. This slope tissue scenario could be further generalized to slopes with other angles of inclination or even an inverted tissue surface, which may be encountered in the clinical setting.

It is worth noting that in teleHRI mode, a regular camera on one side of the slope for side view is mounted with the same angle of inclination as the slope such that the inclined tissue surface in the camera view appears as a horizontal tissue surface. This setting is reasonable since the user is able to use any angle of view for a good viewpoint in *p*HRI mode. Moreover, since the pose mapping algorithm between the robot and the stick is based on relative displacements to their own initial poses, the motion of the stick on a horizontal surface can be automatically mapped to control the motion of the US probe on the inclined slope. This operational flexibility can help the user to obtain a better view and perform comfortable movements on the remote doctor side in teleHRI mode if needed.

**Figure 6:**
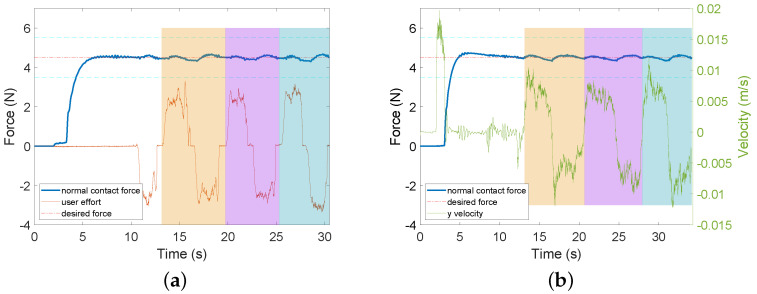
Sample data for US scanning on inclined slope tissue surface with different feedback are provided for two modes in Experiment 2b. AF means audio feedback; HF means haptic feedback; FC means force controller. Two horizontal dashed cyan lines define the tolerance area for the desired force. (**a**) AF + FC + HF (*p*HRI mode). (**b**) AF + FC (teleHRI moce).

A sample of data from Experiment 2b is shown in Figure 6. In Figure 6a, user effort is represented by the user-exerted force along the movement direction of the US probe (i.e., along the slope in this experimental scenario). As can be seen from the figure, user effort in *p*HRI mode is in the range of [−4,4] N, which is relatively small. This means that the human user can easily control the lateral movements of the probe on the slope when an additional force controller is implemented.

Statistical analysis (see Table A4 for details) shows that there is no significant difference (p=0.1412) between the two modes in the mean normal contact force, and also no significant difference (p=0.1504) in their variances.

The results in Experiments 2a and 2b show comparable task performance accuracy and task performance stability in teleHRI mode (with AF + FC) and in *p*HRI mode (with AF + FC + HF), which indicates the potential capability of teleHRI mode to be taken as an alternative for *p*HRI mode even without HF. In addition, compared to Experiment 1, task performance stability in Experiments 2a and 2b is significantly improved (all ps<0.002).

The results in Experiment 2 indicate the same conclusion as that obtained in Experiment 1, i.e., audio feedback can be a good replacement for haptic feedback. More importantly, the hybrid admittance-force controller implemented in Experiment 2 further relieves the need for haptic feedback in teleHRI mode.

### 3.4. Experiment 3: Dual-Mode Switching

Experiment 3 is designed to evaluate the overall performance of the proposed dual-mode *p*HRI-teleHRI control system when mode switching is involved. This experiment requires the human user to perform the task using a “1-2-1-2” sequence, i.e., first to perform the task using the stick (in teleHRI mode) for one session, then perform the task using the robot EE handle (in *p*HRI mode) for another session, then perform the task in teleHRI mode again for one session, then perform the task in *p*HRI mode again. This procedure is repeated another two times in order to generate six sessions for each mode.

**Figure 7:**
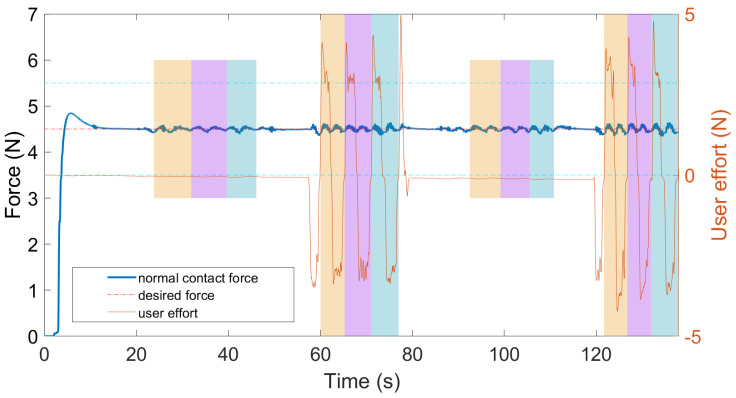
Sample data for dual-mode switching test using a “1-2-1-2” switching sequence in Experiment 3. Each color bar period represents a trial. Two short color bar areas represent the teleHRI mode, while two long color bar areas represent the *p*HRI mode. Two horizontal cyan lines define the tolerance area for the desired force.

The task procedure in this experiment can be better understood via the sample data shown in Figure 7. In the figure, two short bar areas represent the teleHRI mode, while two long bar areas represent the *p*HRI mode. User effort indicates the user-exerted force on the robot EE handle (in *p*HRI mode) along the lateral movement direction of the US probe. As can be seen in the figure, the switching between the *p*HRI and teleHRI mode is seamless, smooth, and stable, and it can be performed whenever necessary, without involving stability issues. This is reasonable and expected due to the relative-displacement-based mapping method, which will be discussed in more detail in the next section.

Statistical analysis (see Table A5 for details) shows that there is no significant difference in the task performance accuracy between the two modes (p=0.1747) in terms of the normal contact force. Although there is a significant difference statistically in their variances (p=0.0033), it is noticed that all the standard deviation values are less than 0.1 N (i.e., less than the F/T sensor resolution). Considering this, it can be safely concluded here that there is no significant difference found between the two modes in terms of either normal contact force or their variances when switching is involved, which indicates the robustness of the proposed dual-mode system during mode switching.

### 3.5. Statistical Comparison across Experiments

The longitudinal comparison of the task performance accuracy and the task performance stability across Experiments 1, 2a, 2b, and 3 is conducted in *p*HRI mode and in teleHRI mode separately by using a t-test. Hereafter, for compactness, EX.1, 2a, 2b, and 3 will be used to represent Experiments 1, 2a, 2b, and 3, respectively.

**Figure 8:**
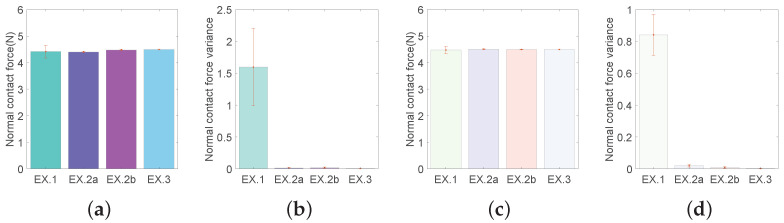
Bar chart for normal contact force and variance. ‘EX.1’, ‘EX.2a’, ‘EX.2b’, ‘EX.3’ represent Experiments 1, 2a, 2b, and 3, respectively. (**a**) Mean in *p*HRI mode. (**b**) Variance in *p*HRI mode. (**c**) Mean in teleHRI mode. (**d**) Variance in teleHRI mode.

In *p*HRI mode, there is no significant difference in the normal contact force between EX.1 and either of the other experiments (see Figure 8a). However, it should be noted that the mean values cannot truly reflect the task performance stability, which mainly depends on their variances. For their variances, there is a significant difference (all ps<0.002) between EX.1 and either of the other experiments in *p*HRI mode (see Figure 8b). Similar statistical results are obtained for teleHRI mode (see Figure 8c,d). These results indicate that both in *p*HRI and teleHRI modes, the task performance is significantly improved in terms of task performance stability and reliability by implementing the designed hybrid admittance-force controller (EX.2a, 2b, 3). The statistical analysis results for the longitudinal comparison across experiments are summarized in Table A6.

## 4. Discussions

In this paper, we propose a dual-mode *p*HRI-teleHRI control system with a custom-designed hybrid admittance-force controller for US imaging. The effectiveness of the proposed system is experimentally evaluated. Experiment 1 is conducted to investigate the possible effects of different feedback types on task performance in two modes, i.e., audio and haptic feedback (AF + HF) in *p*HRI mode and audio-only feedback (AF) in teleHRI mode. Despite the absence of haptic feedback (HF) in teleHRI mode, the task performance is comparable to that in *p*HRI mode. In other words, the audio feedback is capable of being a sufficient replacement for the haptic feedback in our case.

Experiment 2 is an improved version of Experiment 1 while implementing the hybrid admittance-force controller. The results show that, again, the task performance in the mode without haptic feedback (teleHRI) is as good as that in the mode with haptic feedback (*p*HRI). In other words, the audio feedback can be a sufficient alternative to the haptic feedback in this paper. Additionally, the implementation of the hybrid admittance-force controller in Experiment 2 significantly improves the task performance when compared with Experiment 1.

In Experiment 2b with the teleHRI mode, benefiting from the relative-displacement-based mapping method, the movement of the stick on a horizontal surface could be mapped to control the movement of the US probe on an inclined slope surface. In addition, the rotation angle of the regular camera for side view can be tuned as necessary such that a slope surface in the physical world can be shown as a horizontal surface in the camera view. This operational flexibility allows the user to perform comfortable movements and obtain a good view in teleHRI mode if needed. The optional setting for aligning the camera with the slope makes the teleHRI mode comparable to the *p*HRI mode considering that the human user is able to adjust their view point for a better perspective in either mode.

Experiment 3 assessed the overall performance of the proposed dual-mode system when switching mode (i.e., switch *p*HRI mode to teleHRI mode, or vice versa) is involved. The proposed system does not require an actual switch “button” since the two modes are co-existent and coupled via a summation operator to the desired Cartesian pose (in the P-controller in Figure 2). Therefore, in order to perform a switch between the two modes, the user only needs to switch the tool used for the operation, i.e., the robot EE handle for *p*HRI mode or the stick for teleHRI mode. Then, the system will automatically run into the corresponding mode according to the tool used by the user. Due to the relative-displacement-based mapping method, the dual-mode switching is seamless and smooth, and does not involve stability issues. The results also indicated that the dual modes can be switched from one to another at any time point, which can ensure the robustness and safety of the proposed dual-mode system in case of emergency cases. A potential advantage of this dual-mode design is that even during the tele-operation in teleHRI mode, other users (e.g., an assistant) on the patient side can interfere in the ongoing tele-operation whenever necessary and manually move the US probe away from the tissue/patient by using the robot EE handle.

The overall experimental results indicated that the newly designed tele-operation method (teleHRI mode) is capable of being used as an alternative to the *p*HRI method for US scanning when physical distancing is required or when *p*HRI is not available. The capability of allowing seamless switching between the dual modes at any time enables the robustness of the proposed system.

One potential benefit from the proposed system is the low-cost, easy-to-deploy device on the remote doctor side compared with the traditional leader–follower tele-operation systems. High-cost devices has been taken as one main factor inhibiting the implementation of the traditional tele-operation system in the healthcare field [19]. The remote operation method proposed in this paper (teleHRI mode) only needs a tracking camera rather than a sophisticated, expensive, multi-DOF haptic device on the doctor side. Additionally, the cost can be further lowered by choosing a cheaper, regular camera as the tracking device.

Another potential benefit is that it can potentially relieve the strenuous physical efforts and constraints experienced during physical interaction [19] since the stick used on the remote side could be made as light as possible. Moreover, it allows the user to use any available support or any comfortable body posture, thus reducing fatigue. Especially for US scanning tasks that require a long time to complete, this flexibility could be beneficial to sonographers.

One limitation of the proposed system is the low accuracy of the registration between the remote tracking camera frame and the local robot frame. Therefore, the proposed system is not suitable for high-accuracy-demanding tele-operation scenarios.

## 5. Conclusions

In this paper, a dual-mode *p*HRI-teleHRI control system with a hybrid admittance-force controller is developed for US imaging. Instead of employing an expensive and sophisticated robot as a leader on the remote doctor side, a low-cost tracking camera and a stick attached with a tracking marker are utilized to remotely control the robot that is on the patient side. The tele-operation method with only audio feedback (i.e., in teleHRI mode) showed comparable task performance to the physical interaction method with audio and haptic feedback (i.e., in *p*HRI mode). This verified that the designed audio feedback can be a sufficient replacement for haptic feedback in our case, and the teleHRI mode is capable of being used as an alternative method when physical distancing needs to be respected. Furthermore, experimental results showed that the *p*HRI and the teleHRI modes can be switched from one to another seamlessly at any time point without affecting system stability, which demonstrates the robustness and stability of the proposed system.

The dual-mode control system and hybrid admittance-force controller can be easily adapted to other applications where tele-operation is needed beyond the pandemic, such as needle insertion, auscultation, and palpation. In future work, automatic path planning and trajectory tracking, as well as virtual fixture guiding, will be introduced into the system for better task repeatability, which can result in a more intelligent and autonomous system.

## Data Availability

Not applicable.

## Supplementary Materials

The following are available online at https://www.mdpi.com/article/10.3390/s22114025/s1, Video S1: Video demonstration for the experiments on ultrasound imaging with the dual mode *p*HRI-teleHRI control system.

## Appendix A

In this appendix, a set of tables for the experimental results are presented.

**Table A1:** Comparing task performance under different feedback types in two modes when the proposed hybrid controller is NOT implemented in Experiment 1.

		Mean		Variance	
		AF + HF (*p*HRI)	AF (teleHRI)	AF + HF (*p*HRI)	AF (teleHRI)
$F_{z}$ (N)	s1	4.45	4.59	2.1316	0.9409
	s2	4.42	4.46	2.4964	0.6561
	s3	4.55	4.31	0.9025	0.8100
	s4	4.29	4.35	1.5129	0.8464
	s5	4.76	4.69	1.1664	1.0404
	s6	4.03	4.45	1.3225	0.7744
		p=0.5457		p=0.0420 (*)	

Note: s1~s6 represent session 1~6, respectively; AF means audio feedback; HF means haptic feedback; *Fz* means normal contact force; * means significance level under 5%.

**Table A2:** Time percentage for keeping force within desired range under different feedback types in two modes when the proposed hybrid controller is NOT implemented in Experiment 1.

		Trial 1	Trial 2	Trial 3	Mean ± Std.
*p*HRI mode (%)	s1	55.36	51.26	47.48	51.37±3.94
	s2	63.52	64.64	35.83	54.66±16.32
	s3	62.54	72.51	75.54	70.20±6.80
	s4	62.87	63.05	46.68	57.53±9.40
	s5	66.73	50.50	75.56	64.26±12.71
	s6	53.78	59.49	50.15	54.47±4.71
		58.75±10.73 (mean ± std.)			
teleHRI mode (%)	s1	73.91	70.40	79.09	74.47±4.37
	s2	75.46	70.79	91.51	79.25±10.87
	s3	63.92	73.29	74.15	70.45±5.67
	s4	64.49	72.29	80.27	72.35±7.89
	s5	78.11	69.44	68.63	72.06±5.25
	s6	76.31	75.85	80.72	77.63±2.69
		74.37±6.47 (mean ± std.)			

Note: s1~s6 represent session 1~session 6, respectively; std. means standard deviation.

**Table A3:** Comparing the task performance on horizontal tissue surface under different feedback types in two modes when the proposed hybrid controller is implemented in Experiment 2a.

		Mean		Variance	
		AF + FC + HF (*p*HRI)	AF + FC (teleHRI)	AF + FC + HF (*p*HRI)	AF + FC (teleHRI)
$F_{z}$ (N)	s1	4.43	4.53	0.0064	0.0121
	s2	4.41	4.51	0.0036	0.0100
	s3	4.41	4.50	0.0144	0.0225
	s4	4.42	4.49	0.0100	0.0169
	s5	4.36	4.51	0.0100	0.0324
	s6	4.39	4.52	0.0324	0.0225
		$p=2.6999\times 10^{-4}$ (*)		p=0.1755	

Note: s1~s6 represent session 1~6, respectively; AF means audio feedback; HF means haptic feedback; FC means force controller; *Fz* means normal contact force; * means significance level under 5%.

**Table A4:** Comparing the task performance on slope tissue surface under different feedback types in two modes when the proposed hybrid controller is implemented in Experiment 2b.

		Mean		Variance	
		AF + FC + HF *p*HRI)	AF + FC (teleHRI)	AF + FC + HF (*p*HRI)	AF + FC (teleHRI)
$F_{z}$ (N)	s1	4.49	4.51	0.0289	0.0036
	s2	4.43	4.49	0.0144	0.0025
	s3	4.49	4.50	0.0144	0.0121
	s4	4.49	4.50	0.0121	0.0064
	s5	4.49	4.50	0.0361	0.0081
	s6	4.50	4.49	0.0081	0.0196
		p=0.1412		p=0.1504	

Note: s1~s6 represent session 1~6, respectively; AF means audio feedback; HF means haptic feedback; FC means force controller; *Fz* means normal contact force.

**Table A5:** Comparing the general performance of the proposed dual-mode *p*HRI-teleHRI system when mode switching is involved in Experiment 3.

		Mean		Variance	
		*p*HRI	teleHRI	*p*HRI	teleHRI
$F_{z}$ (N)	s1	4.50	4.50	0.0025	0.0016
	s2	4.49	4.50	0.0049	0.0016
	s3	4.50	4.50	0.0036	0.0025
	s4	4.49	4.50	0.0064	0.0036
	s5	4.50	4.50	0.0049	0.0016
	s6	4.50	4.50	0.0049	0.0016
		p=0.1747		p=0.0033 (*)	

Note: s1~s6 represent session 1~6, respectively; *Fz* means normal contact force; * means significance level under 5%.

**Table A6:** Summary of *p*-values of *t*-test results.

		EX.2a	EX.2b	EX.3	EX.2a	EX.2b	EX.3
		Mean			Variance		
EX.1	$F_{z}$ (*p*HRI)	0.9031	0.5530	0.4600	0.0015 *	0.0014 *	0.0014 *
EX.2a		-	0.0042 *	0.0004 *	-	0.4436	0.1035
EX.2b		-	-	0.1647	-	-	0.0276 *
EX.1	$F_{z}$ (teleHRI)	0.5546	0.7033	0.6876	0.00002 *	0.00002 *	0.00002 *
EX.2a		-	0.1099	0.1438	-	0.0152 *	0.0037 *
EX.2b		-	-	0.6109	-	-	0.0529

Note: EX.1, 2a, 2b, 3 mean Experiment 1, 2a, 2b, 3, respectively; *Fz* means normal contact force; * means significance level under 5%.
